# Osteoporosis Self-Assessment Tool for Asians Can Predict Neurologic Prognosis in Patients with Isolated Moderate Traumatic Brain Injury

**DOI:** 10.1371/journal.pone.0132685

**Published:** 2015-07-17

**Authors:** Chia-Hung Chao, Yu-Feng Su, Hon-Man Chan, Shiuh-Lin Huang, Chih-Lung Lin, Aij-Lie Kwan, Yun-Ting Lou, Chao-Wen Chen

**Affiliations:** 1 Division of Neurosurgery, Department of Surgery, Kaohsiung Medical University Hospital, Kaohsiung Medical University, Kaohsiung, Taiwan; 2 Graduate Institute of Medicine, College of Medicine, Kaohsiung Medical University, Kaohsiung, Taiwan; 3 Department of Surgery, Faculty of Medicine, College of Medicine, Kaohsiung Medical University, Kaohsiung, Taiwan; 4 Department of Ophthalmology, E-DA Hospital, Kaohsiung, I-Shou University, Kaohsiung, Taiwan; 5 Division of Traumatology, Department of Surgery, Kaohsiung Medical University Hospital, Kaohsiung Medical University, Kaohsiung, Taiwan; 6 Department of Emergency Medicine, Faculty of Medicine, College of Medicine, Kaohsiung Medical University, Kaohsiung, Taiwan; Texas Tech University Health Science Centers, UNITED STATES

## Abstract

**Objectives:**

Osteoporosis Self-Assessment Tool for Asians (OSTA) has been proved to be a simple and effective tool for recognizing osteoporosis risk. Our previous study has demonstrated that the preoperative OSTA index was a good prognostic predictor for stage II and III colon cancer patients after surgery. We aim to evaluate the value of OSTA index in prognostication of isolated traumatic brain injury with moderate severity (GCS 9-13).

**Methods:**

We retrospectively reviewed all patients visiting Kaohsiung Medical University Hospital emergency department due to isolated moderate traumatic brain injury from Jan. 2010 to Dec. 2012. Background data (including the OSTA index), clinical presentations, management and outcomes (ICU admission days, total admission days, complications, Glasgow outcome score (GOS) at discharge, mortality) of the patients were recorded for further analysis. Our major outcome was good neurologic recovery defined as GOS of 5. Pearson chi-square test and the Mann-Whitney U test were used to compare demographic features. Multiple logistic regression was used to identify independent risk factors.

**Results:**

107 isolated moderate TBI patients were studied. 40 patients (37.4%) showed good recovery and 10 (9.3%) died at discharge. The univariate analysis revealed that younger age, higher OSTA index, lower ISS, lower AIS-H, and avoidance to neurosurgery were associated with better neurologic outcome for all moderate TBI patients. Multivariate analysis revealed that lower ISS, higher OSTA, and the avoidance of neurosurgery were independent risk factors predicting good neurologic recovery.

**Conclusion:**

Higher ISS, lower OSTA index and exposure to neurosurgery were the independent risk factors for poorer recovery from isolated moderate TBI. In addition to labeling the cohort harboring osteoporotic risk, OSTA index could predict neurologic prognosis in patients with isolated moderate traumatic brain injury.

## Introduction

Traumatic brain injury (TBI) is a disease harboring significantly injurious impact on society and individuals. Many previous studies have established or validated prognostic models of TBI. The largest two databases were the International Mission on Prognosis and Analysis of Clinical Trials in TBI (IMPACT) [1] and Corticosteroid Randomization After Significant Head Injury trial data (CRASH models)[2,3]. The attributes in IMPACT core models include age, GCS motor scores, pupillary reactivity, CT Marshall scores, and secondary insults, while the CRASH predictors include countries (high or low income), age, GCS, pupil reactivity, extra-cranial injuries, and CT scans [1,2]. Recently, novel prognostic factors of TBI have been studied in animal experiments or clinical trials, such as estrogen, progesterone, vitamin D, etc [4–10]. Interestingly, many of these factors were also the contributing factors of osteoporosis [10–14]. It seems reasonable to hypothesize osteoporosis may be related to TBI prognosis. Nevertheless, the relationship between osteoporosis risk and TBI outcomes has been rarely investigated.

Osteoporosis is a common disorder among aged people, especially post-menopausal women. It increases fracture risks causing substantial morbidity and mortality, and also generates tremendous healthcare burden [15,16]. Bone mineral density measurement using dual‐energy X‐ray absorptiometry (DXA) is the standard diagnostic tool, but requires special instruments that are not always easily accessible [17]. Several screen tools undergone extensive validation have been developed to predict low BMD and fracture risks, such as Age Body Size No Estrogen (ABONE), Body Weight Criterion (BWC), National Osteoporosis Foundation (NOF), Osteoporosis Prescreening Risk Assessment (OPERA), Osteoporosis Risk Assessment Instrument (ORAI), Osteoporosis Index of Risk (OSIRIS), Osteoporosis Self‐Assessment Tool (OST), Osteoporosis Screening Tool for Asians (OSTA), and Simple Calculated Risk Estimation Score (SCORE) [18–23]. Osteoporosis Self-Assessment Tool (OST)/ Osteoporosis Screening Tool for Asians (OSTA) have been externally validated in 19 studies and the sensitivity and specificity ranges from 84% to 97% and 34% to 70% respectively [18,24–27]. In a comparative systemic review, OST/OSTA is the simplest and does better than other complex tools [19,20]. It was first developed by Koh et al., focusing on the postmenopausal female population [28]. Liu et al. also evaluated the diagnostic value of OSTA in aged men and found good correlation between the OSTA index and BMD [29]. Fransiska et al. evaluated OSTA in Indonesian men, and its sensitivity and specificity were 74% and 41% respectively [30]. In a recent study, we identified the pre-operative OSTA index as the robust predictor of cancer-specific mortality in stage II / III colon-rectal cancer after surgery [31]. This was the first study to investigate the association between the OSTA index and outcome of chronic illness. We hypothesized that risk assessment of osteoporosis may be as a proxy of recognition of physical frailty. Accordingly, in the present study, we intended to validate a risk assessment tool of osteoporosis for the prognostic prediction of traumatic brain injury.

## Methods

### Study Design

This is a retrospective cohort study based on prospectively collected trauma registry and acute care surveillance data in a tertiary care hospital. The study patient records/information was anonymized and de-identified prior to analysis. This study was approved by the hospital’s Institutional Review Board. (IRB-20130018)

### Patient Cohort

Based on our previous study, we found osteoporosis might generate prognostic impact on cancer patients with moderate disease severity [31]. We proposed that cancer offence is negligible in stage I patients and overwhelming in stage IV patients (one-sided game theory). Based on this concept, we merely focus on moderate traumatic brain injury patients (GCS: 9–13) as our study population. We believe that this particular design would provide more conclusive evidence relevant to the OSTA effect on TBI outcome after excluding the patients whose injury severity was too mild or severe. From January 2010 to December 2012, all adult traumatized patients (aged above 16 years) presented to the emergency department (ED) of Kaohsiung Medical University Hospital for first aid were initially reviewed. According to the management principle of TBI in our institution, brain CT examination was arranged after vital signs were stabilized according to ATLS guidelines. Patients who met admission criteria were admitted to the neurosurgical intensive care unit, neurosurgical ward, or trauma ward according to disease severity. Admission criteria include patients with intracranial hemorrhage (either or not receiving neurosurgical intervention), poor GCS scores, diffuse axonal injury, or intolerable post-concussion syndrome. Furthermore, we planned to enroll isolated TBI patients in order to expel the potential influence of other associated injuries on each patient’s outcome. Those who had associated extra-cranial injuries with significant severity (AIS score of body parts other than head and neck more than 2 points) were excluded. Patients with moderate TBI who did not receive brain CT survey or failed to meet the admission criteria were also excluded for their incomplete acquisition of clinical information.

### Data Collection

We reviewed all qualified patients and retrieved clinical information including individual background data (age, weight, sex, underlying diseases), clinical presentations (Injury Severity Score, New Injury Severity Score, Abbreviated injury score of head, GCS score, CT grading according to the Marshall classifications), treatment of TBI (avoidance or exposure to neurosurgical management relevant to TBI), and outcomes (mortality in hospital, total length of stay {LOS}, LOS in intensive care unit {ICU}, presence of complications noted by trauma registry, and Glasgow outcome score {GOS} at discharge). OSTA index can be calculated using the formula of (weight in kilograms–age in years) × 0.2. All the OSTA-related information on each patient was collected during the admission.

### Outcome Measurement

The primary outcome measurement was recovery from traumatic events according to GOS. This is a well-validated measurement scale with scores varying from 1 to 5 [32]. We defined good recovery as GOS of 5, and inadequate recovery as a score of 1–4. To ensure consistent outcome evaluations and to avoid conflicts of interest, the GOS at discharge was determined by two different authors while blinded to all clinical data. In addition, according to trauma registry reports, presence or absence of complications developed during admission was also treated as secondary outcomes.

### Statistical Analysis

All parameters in the present study were evaluated for normality by applying the Kolmogorov-Smirnov test. The data are presented as median (interquartile range {IQR}) when the normality assumption is violated. Pearson chi-square test and the Mann-Whitney U test were used to compare demographic features among groups. Multiple stepwise regression analyses were performed to identify variables that were significantly related to the likelihood of major outcome (Good recovery, GOS = 5) and secondary outcomes (complications). Regression models were controlled for the effects of confounding variables. Results of the logistic regression analysis were reported as adjusted odds ratio (OR) with 95% confidence interval (CI). The Receiver Operating Characteristic (ROC) curves were plotted for predictors, and the area under the curve (AUC) was used to evaluate the predictive value. All tests of significance were two tailed, and P-values < 0.05 were considered statistically significant. The data were analyzed with Statistical Package for the Social Sciences Version 16.0 (SPSS. Inc., Chicago, IL).

## Results

### Characteristics of the Study Cohort

**Fig 1:**
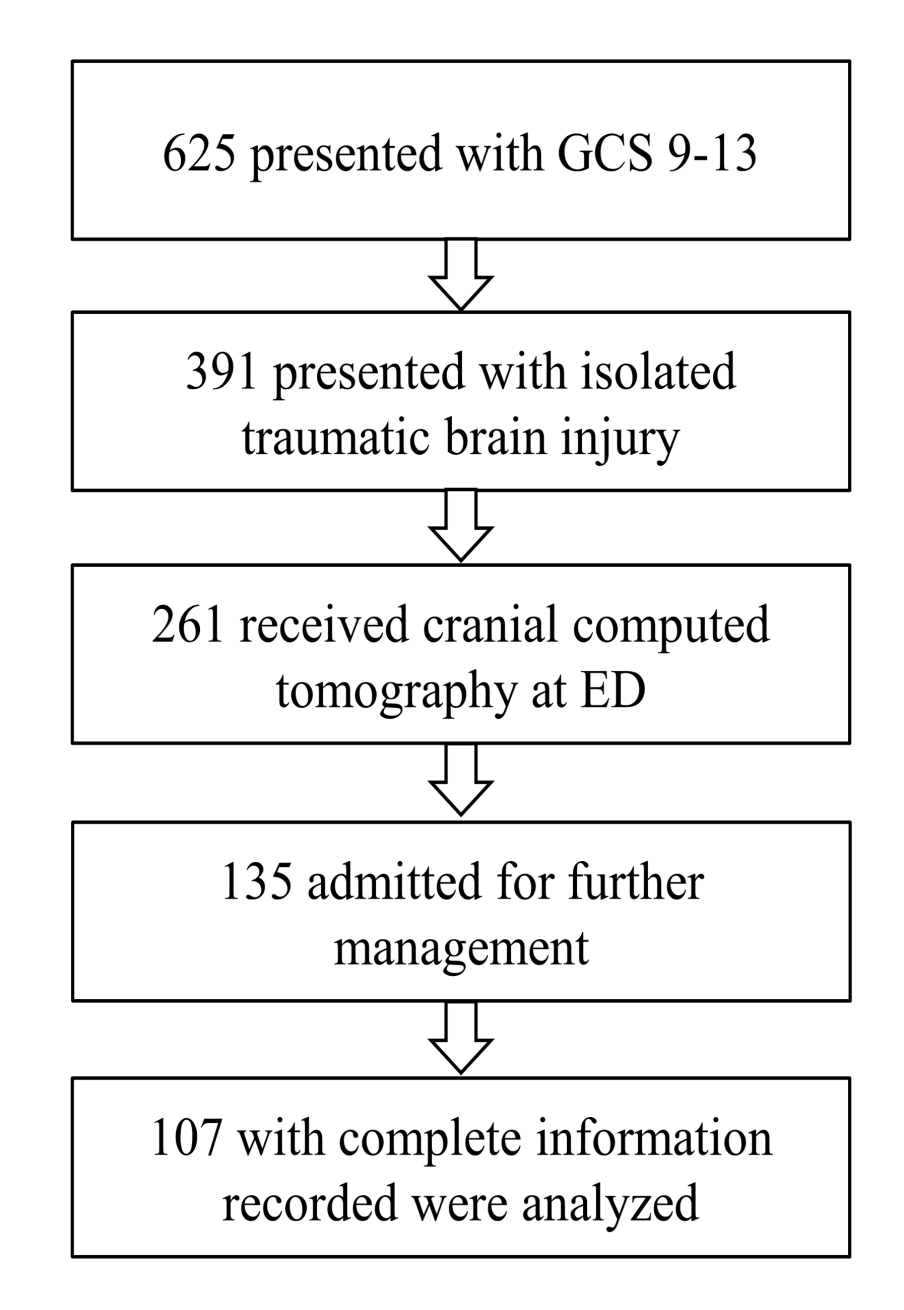
Algorithm for study case selection.

**Fig 2:**
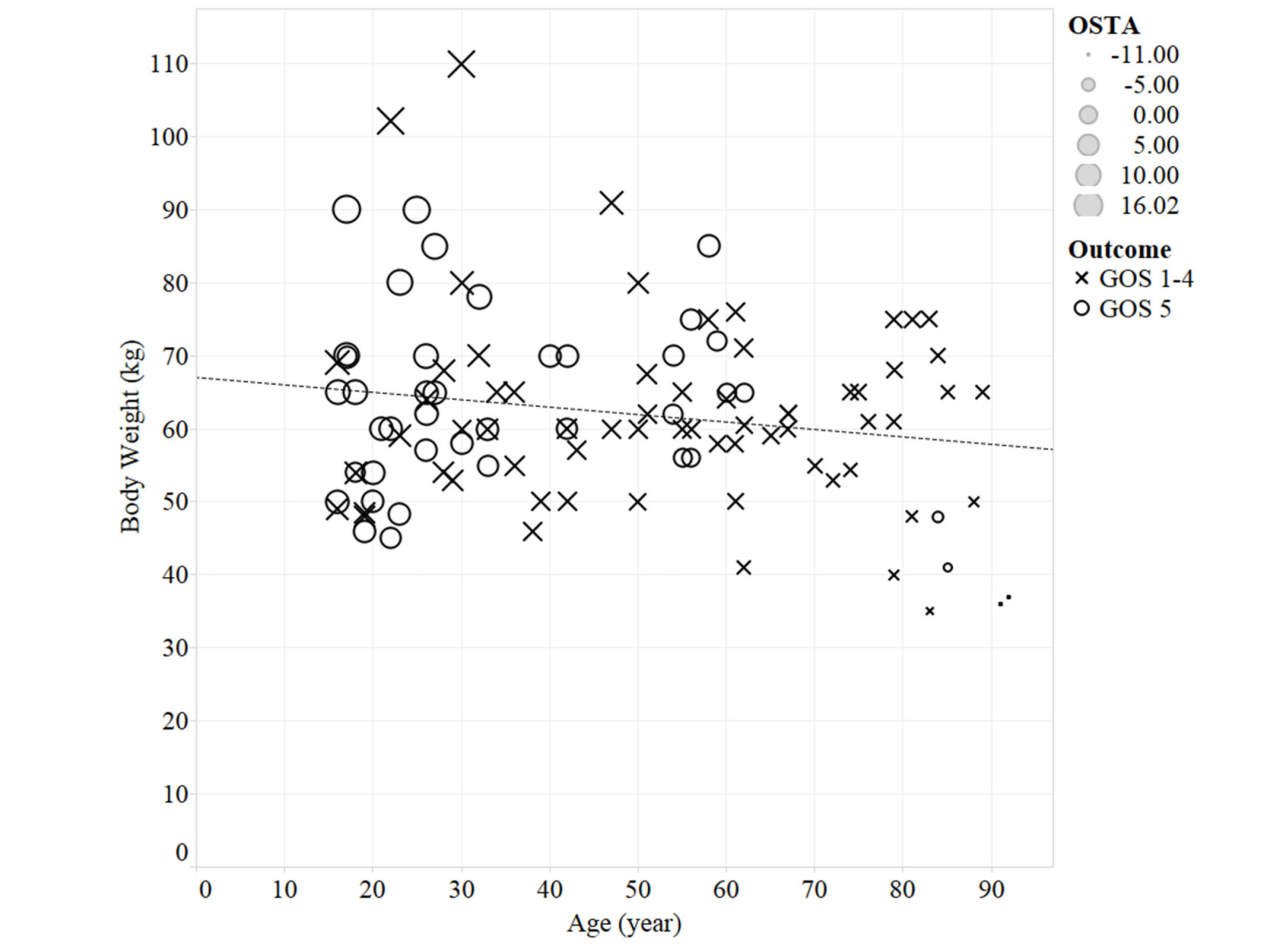
Scatter plot of Body weight and age both labelled by OSTA undex. The scatter plot displayed no significant correlation between body weight and age in our study cohort. (Pearson’s r = -0.064, *p* = 0.515). The size of the symbol indicates the OSTA value.

During the 3-year study period, 135 of 625 moderate TBI patients presenting to our emergency department were admitted for further management. 107 isolated moderate TBI with complete clinical information were included in our study cohort. (Fig 1) The median age was 47 (IQR 26–62) years, and the median body weight was 60 kg (IQR 53–69). The mean OSTA index was 2.8±5.6. The association of these three variables is shown in Fig 2. The majority of the study cohort was male (62/107, 57.9%). The median Injury Severity Score (ISS), Abbreviated injury severity score of head (AISH) and GCS at admission were 16 (IQR 9–17), 4 (IQR 3–4), and 11 (IQR 10–12), respectively. The initial brain CT grade of the patients were grade 1–2 (76, 71%), grade 3–4 (19, 17.8%), grade 5–6 (12, 11.2%). There were 32 patents (29.9%) receiving Neurosurgery. The median hospital LOS was 9 days (IQR 5–19), and the median ICU stay was 3 days (IQR 0–7). 34 of 107 (31.8%) patients had complications developed during admission. Most patients recovered well with a GOS of 5 (40, 37.4%), followed by GOS 4 (32, 29.9%), GOS 3 (22, 20.6%), GOS 1 (10, 9.3%), and GOS 2 (3, 2.8%) respectively. Ten patients died (a mortality rate of 9.3%).

### Outcome Analysis

**Fig 3:**
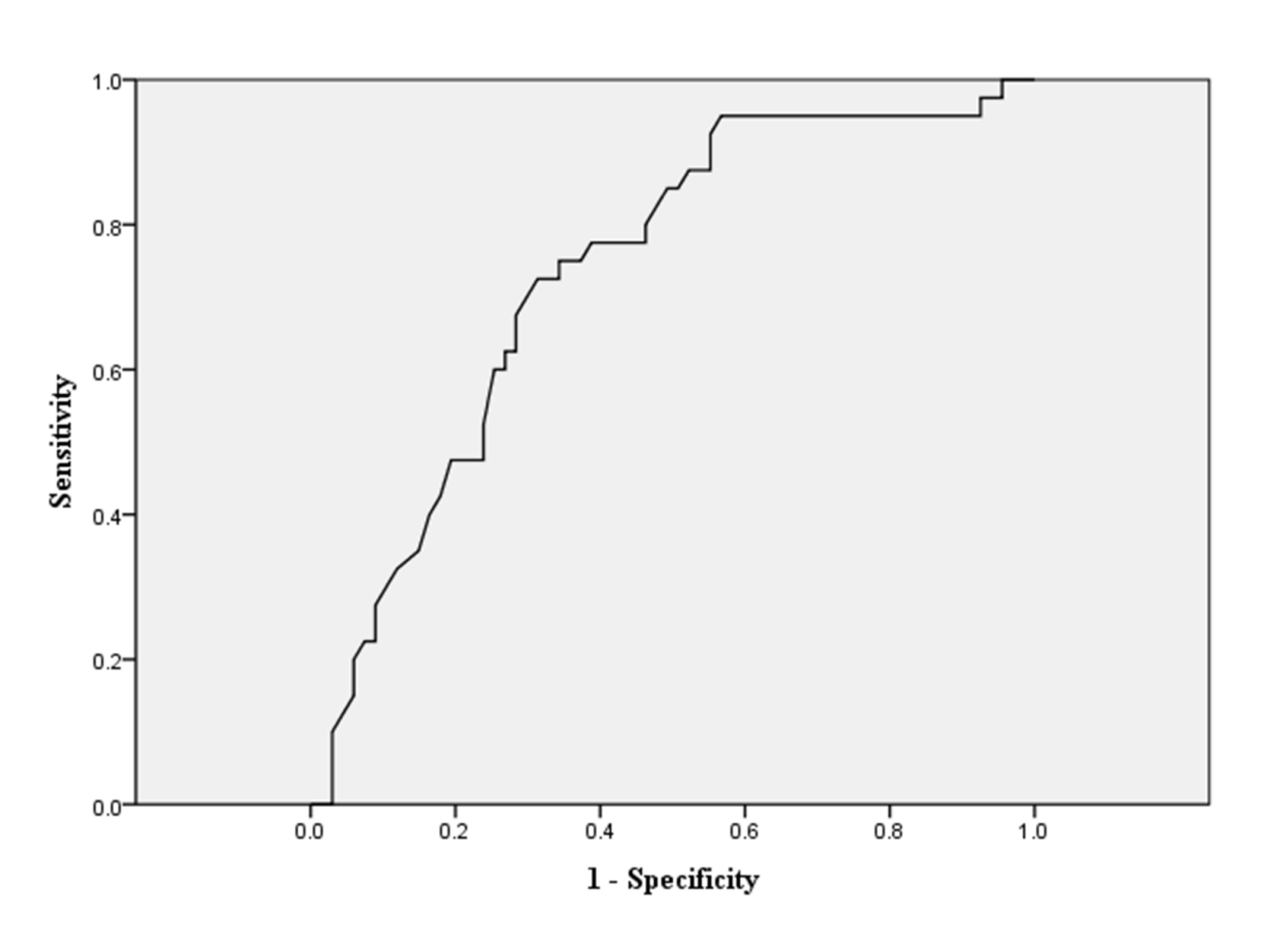
Diagnostic performance of the OSTA index for good neurologic recovery (GOS = 5) in the study cohort. Receiver Operating Characteristic (ROC) curve shows an area of 0.734 (95% CI 0.638–0.831).

**Fig 4:**
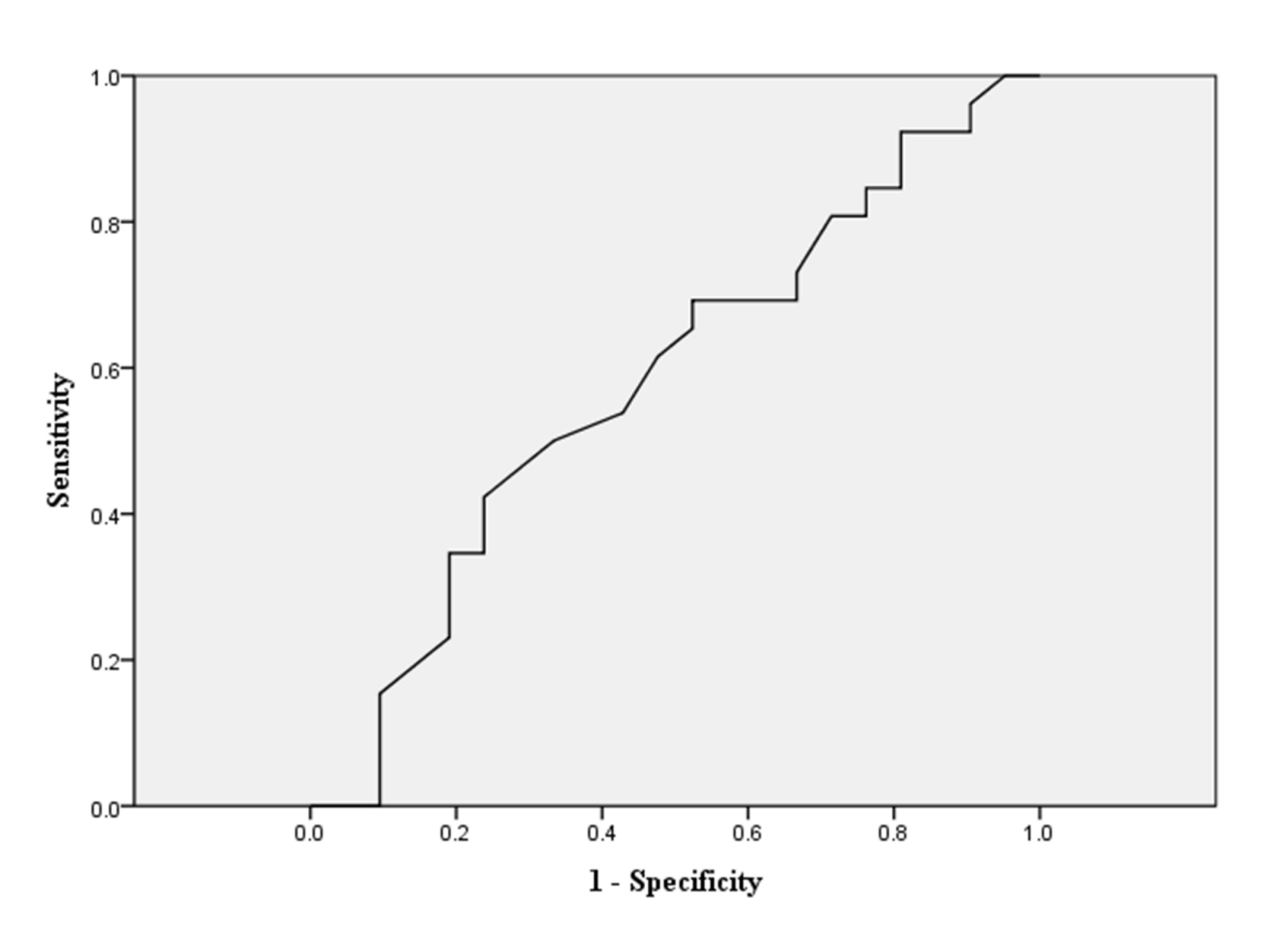
The ROC curve for the OSTA index predicting good neurologic recovery in the younger group (age<40). ROC curve of OSTA index with respect to detecting younger patient with GOS 5 with an AUC of 0.582.

**Fig 5:**
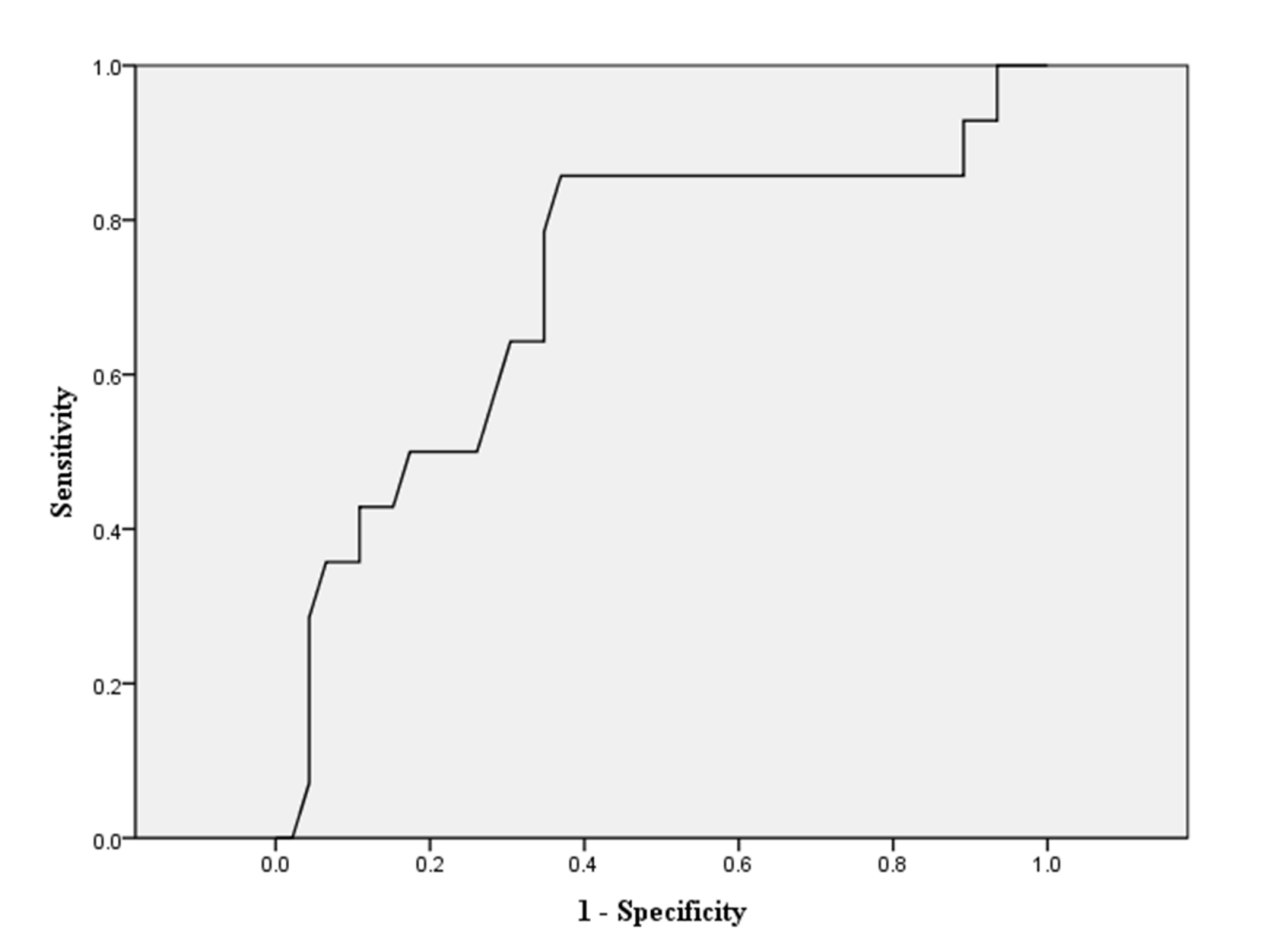
The ROC curve for the OSTA index predicting good neurologic recovery in the older group (age≧40). ROC curve of OSTA index with respect to detecting GOS 1–4 with an AUC of 0.719.

As our previous description, we dichotomized all cohorts into good recovery group (GOS 5) and inadequate recovery group (GOS 1–4). The related demographic information is shown in Table 1. In the univariate analysis, younger age, higher OSTA index, lower ISS and NISS, lower AIS-H, and avoidance to neurosurgery were associated with better outcome for all moderate TBI patients (all *p*-value<0.001). We further conducted multivariate logistic regressions to identify the independent influential factors. In Table 2, we found that lower ISS, higher OSTA, and the avoidance of neurosurgery were the independent risk factors predicting good recovery. As the original OSTA index was retrieved and validated from older Asian populations (age≧40), we decided to conduct the age-stratified analysis for elucidating more findings. We initially dichotomized all cohorts into younger group (age<40) and older group (age≧40), and then performed the multivariate logistic regressions analysis again. (Table 2) In the younger group, the need of neurosurgery appeared to be the only independent risk factor for poor outcome. On the other hand, in the older group, lower ISS and higher OSTA index were demonstrated to be independent risk factors for good recovery. The sensitivity and specificity of the OSTA index in relation to good recovery were plotted as receiver-operating characteristic (ROC) curves. The areas under the curves (AUC) were calculated and the OSTA index provided AUC of 0.734 (Fig 3). Regarding the younger group, the ROC analysis showed that OSTA index was incapable of recognizing moderate TBI patients with good recovery (Fig 4). Nevertheless, the discriminative value was still acceptable in the older group with an AUC of 0.719. (Fig 5). Regarding the secondary outcome, defined as presence of complications, the multivariate logistic regression revealed age and ISS were two independent factors that significantly influenced complications rates. The odds ratios were 1.05 and 1.24 (*p*<0.001) respectively. (Table 3) OSTA index generated no significant influence on secondary outcome.

**Table 1:** Univariate analysis regarding demographic and clinical characteristics in GOS-5 and GOS 1–4 groups.

	GOS 1–4 (n = 67)		GOS 5 (n = 40)		
Characteristics	Mean (SD) / Median (IQR)	N (%)	Mean (SD) / Median (IQR)	N (%)	*P*-value*
Male		41 (61.2)		21 (52.5)	0.378
Age (year)	56 (34–74)		33.7 (27, 36)		<0.001
Body Weight (kg)	60 (53–67)		62 (54–70)		0.468
OSTA index	1.29 (5.54)		5.34 (4.69)		<0.001
GCS	11 (10–12)		11 (10–13)		0.189
ISS	16 (9–20)		9 (4–11)		<0.001
NISS	16 (9–20)		9 (9–13)		<0.001
AIS-H	4 (3–4)		3 (2–3)		<0.001
LOS in ICU (day)	5 (2–13)		0.5 (0–3.75)		<0.001
LOS in hospital (day)	14 (7–26)		5.5 (3–8.75)		<0.001
Receiving Neurosurgery		30(44.8)		2(5)	<0.001

*The gender and receiving neurosurgery variables were tested by Chi-square test, the OSTA variable was tested by student-*t* test, and the other variables were tested by Mann-Whitney U test.

**Table 2:** Independent factors for primary outcome in the multivariate analyses regarding different study cohorts.

Variable	Odds Ratio (95% CI)*	*P*- value
All cohorts (n = 107)		
ISS	0.869 (0.781–0.960)	0.005
OSTA	1.157 (1.057–1.266)	0.002
Exposure to neurosurgery	0.162 (0.031–0.846)	0.031
Younger group (Age<40, n = 47)		
Exposure to neurosurgery	0.135 (0.025–0.734)	0.020
Older group (Age≧40, n = 60)		
ISS	0.601 (0.427–0.845)	0.003
OSTA	1.753 (1.199–2.564)	0.004

*Adjusted for gender, age, body weight, OSTA index, GCS, ISS, NISS, AIS-H and exposure of neurosurgery.

**Table 3:** Independent factors for secondary outcome (complications) in the multivariate analyses.

Variable	Odds Ratio (95% CI)*	*P*- value
All cohorts (n = 107)		
Age	1.050 (1.204–1.076)	<0.001
ISS	1.235 (1.098–1.390)	<0.001

*Adjusted for gender, age, body weight, OSTA index, GCS, ISS, NISS, AIS-H and exposure of neurosurgery.

## Discussion

To the best of our knowledge, our study is the first study to evaluate the association between osteoporotic risks and prognosis of acute trauma brain injury. With risk adjustment, higher ISS score, lower OSTA index and exposure to neurosurgery were independent factors predicting poorer Glasgow outcome scores in patients with isolated moderate TBI. Furthermore, in the age-stratified analysis, ISS and OSTA appeared to be the only two independent risk factors for the older cohort. Although body weight and age are main components of the OSTA index, neither of them yielded statistical significance in our multivariate analysis. Regarding these independent influential factors, the prognostication of ISS score has been well established before [33–37]. On the other hand, the OSTA index is a good representative for osteoporotic risks, which have been externally validated with bone mineral densities among many kinds of Asian populations [24,25,27–30]. The OSTA index only considers the age and the body weight as variables and it is a simple and convenient tool to estimate osteoporotic risks for physicians and patients. Our results infer that the OSTA index might act as a proxy of body fragility. Just like the two sides of a coin, ISS means external offense and OSTA means internal defense while encountering acute trauma.

**Fig 6:**
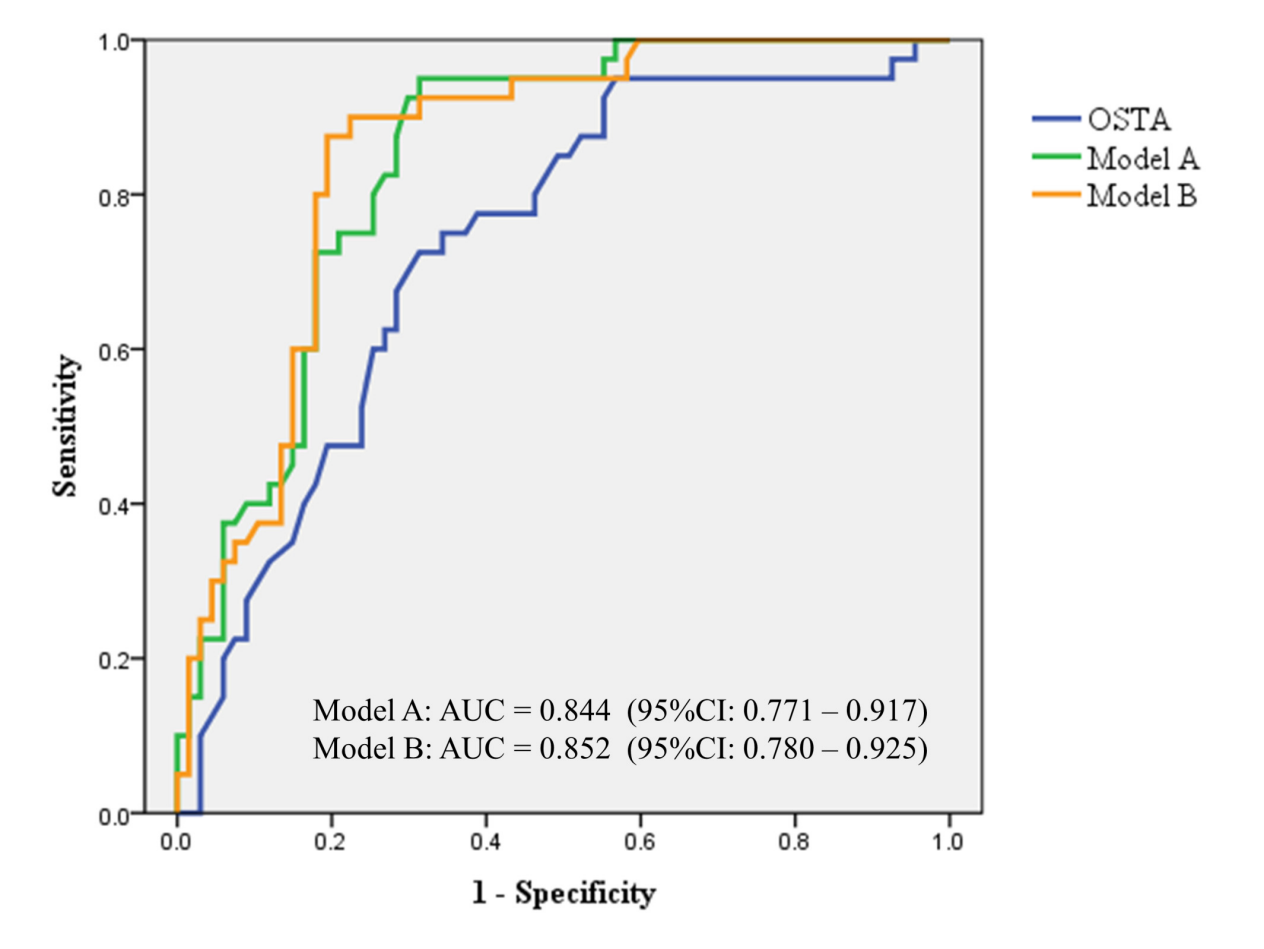
The ROC curves comparing the proposed models and the OSTA index to predict good neurologic recovery in the study cohort. The discriminative performance of model B (ISS, OSTA index, and exposure to neurosurgery) is superior to model A (ISS and OSTA index) and OSTA index alone.

According to the original definition, OSTA index >−1 is classified as having low risk of osteoporosis, −1 to −4 as intermediate risk, and <−4 as high risk.[23,24,28,38] Nevertheless, we did not introduce the polychotomous variables into our analysis as we worked on evaluating the clinical implications of the OSTA index on acute injury prognosis instead of exploring the association of osteoporosis risk. We hypothesized that there would be a different cut-off value of the OSTA index capable to distinguish those with good recovery from the moderate TBI cohort. In the present study, the optimal threshold of the OSTA index was evaluated by maximizing the sum of sensitivity and specificity. The threshold of 3.5 was identified with the corresponding AUC of 0.734. In order to improve the prognostication capability of GOS outcome, the probability of the multivariate logistic regression model using the three independent factors including exposure to neurosurgery, ISS, and OSTA index was calculated. We introduced two prediction models: model A including ISS and OSTA index, and model B including ISS, OSTA index, and exposure to neurosurgery. In the comparison analysis of different predictive models, the discriminative power of the model B was superior to other models. (Fig 6) However, the difference between model A and B was not obvious. (AUC = 0.844 vs. o.852 respectively; *p*-value = 0.88) If we merely take ISS and OSTA index into our consideration for performing the linear regression analysis, we could conduct a formula integrated with standard regression coefficients: “(0.32 x OSTA)–(0.52 x ISS)” and then identify a cut-off value of -6.78 with a sensitivity of 0.925 and specificity of 0.701. Compared to other clinical predictors derived from other relatively heterogeneous cohorts, our model focusing on a homogenous group (isolated moderate TBI) could generate a pertinent and less confounding conclusion. It also demonstrates some strengths including intuitive concept, rapid implementation into clinical practice, and limited utilization of resources. We propose the composite score incorporating ISS and OSTA index as a rapid prognostic predictor while approaching moderate TBI patients.

The linkage between osteoporosis risk and traumatic outcome still warrants more investigations. Gan et al.[39] performed a retrospective cohort study of 324 patients with a standardized protocol for treatment of moderate and severe head injury, and the author concluded age was an independent factor in the outcome prediction. Bazarian et al.[4] had studied sex differences after mild traumatic brain injury and concluded the female sex was associated with the odds of poorer outcome as post-concussive symptoms scores, and the observed pattern of peak is the childbearing years. The author hypothesized that it may be due to disruption of endogenous estrogen and progesterone production. In a review article, Simpkins et al. had summarized estrogen had a cytoprotective role in the mitochondria against acute brain injury and chronic neuro-degeneration.[5] Estrogen plays a vital role in regulating bone metabolism, and its deficiency causes post-menopausal osteoporosis.[6] Insufficient serum levels of 25-hydroxyvitamin D is also a risk factor for osteoporosis and osteoporotic fractures.[10] The combination of vitamin D and progesterone has been promoted with better outcome in the treatment of traumatic brain injury patients.[9] Chabas et al. performed an animal genetics study and demonstrated vitamin D improves myelination via myelin-associated genes after nerve injury, although the experiment is focusing on the peripheral nerves.[8] Also according to Cekic et al., traumatic brain injury may lead to over-activate defensive responses, and vitamin D is the stabilizing factor of nerve damage and systemic inflammations.[7] Above all, the recovery from traumatic brain injury depends on multiple factors, such as age, estrogen, vitamin D, etc, that are in common with the risks factors of osteoporosis.[11,14] The process of neurologic recovery might be influenced by numerous tiny alterations in a unique host microenvironment. We believe the impact of osteoporosis on acute trauma prognostication is underestimated, and prefer taking our study as a part for understanding and exploring more relationships between osteoporosis and traumatic pathophysiology. In line with our study result, we consider the OSTA index, as a surrogate of frailty, can be used to predict the outcome of traumatic brain injury once the impact of injury itself is not too large or small to overwhelm the outcome.

### Limitations of this Study

Our study still has several limitations. First, the number of study participants is limited. For expelling the prognostic effects caused by other injuries, we only enrolled isolated head trauma patients. Moreover, we excluded those with mild or severe TBI. The excluded portion might possess some impact on the overall results if they were completely enrolled. Second, there is insufficient information regarding the long-term GOS outcome, which might be different from short-term results. Lastly, the need for neurosurgery with relevant outcome might be influenced by different surgeons’ judgment and expertise. A large-scale study of prospective design for controlling potential confounders might alleviate these weaknesses.

## Conclusion

Higher ISS, lower OSTA index and exposure to neurosurgery are the independent risk factors for poorer recovery from isolated moderate traumatic brain injury. The OSTA index could provide additional merit in traumatic prognostication in addition to labelling the cohort harboring osteoporotic risk.

## Supporting Information

S1 Dataset Detailed data relevant to the study. (XLSX)
