# Correction: Osteoporosis Self-Assessment Tool for Asians Can Predict Neurologic Prognosis in Patients with Isolated Moderate Traumatic Brain Injury

**DOI:** 10.1371/journal.pone.0198986

**Published:** 2018-06-11

**Authors:** Chia-Hung Chao, Yu-Feng Su, Hon-Man Chan, Shiuh-Lin Huang, Chih-Lung Lin, Aij-Lie Kwan, Yun-Ting Lou, Chao-Wen Chen

An affiliation for the 2nd and 5th authors is not indicated. Yu-Feng Su and Chih-Lung Lin are both affiliated with: Graduate Institute of Clinical Medicine, College of Medicine, Kaohsiung Medical University, Kaohsiung, Taiwan.
